# Impact of Temperature Stresses on Wheat Quality: A Focus on Starch and Protein Composition

**DOI:** 10.3390/foods14132178

**Published:** 2025-06-22

**Authors:** Pei Han, Yaping Wang, Hui Sun

**Affiliations:** 1 School of Health Science and Engineering, University of Shanghai for Science and Technology, Shanghai 200093, China; hanpeihjt@126.com; 2 Academy of National Food and Strategic Reserves Administration, Beijing 100037, China; sh@ags.ac.cn

**Keywords:** heat stress, cold stress, SSP, amylose, amylopectin, starch synthesis enzymes, wheat grain development, processing quality

## Abstract

With climate change, maintaining wheat quality has become essential for the functional properties, end-use, commodity value, and nutritional benefits of wheat flour. Temperature indirectly influences wheat quality by modulating grain size, starch and protein content, and the balance between these components. This review systematically analyzes temperature-mediated alterations in wheat grain quality, with particular emphasis on the two core components: starch and protein. Specifically, daytime warming generally increases protein content while reducing starch accumulation; however, temperatures exceeding 30 °C diminish key protein quality parameters (UPP%, Glu/Gli ratio, HMW-GS/LMW-GS ratio). Nighttime warming enhances protein quality but compromises starch content and yield potential. Conversely, under low-temperature conditions, starch content declines, whereas protein content is primarily influenced by genotypes and treated temperatures. Furthermore, the underlying mechanisms driving temperature-induced changes in wheat quality traits are discussed. However, the mechanisms of temperature effects have not been fully elucidated, and the results often vary between regions or over years. Thus, identifying conserved high/low-temperature resistance genes, QTLs, epialleles, and epiQTL, as well as developing corresponding molecular markers and epi-markers, is an urgent priority. Meanwhile, genome-editing tools such as CRISPR/Cas could serve as a powerful approach for creating new wheat germplasm with durable high/low-temperature resistance.

## 1. Introduction

As one of the major global food crops, wheat plays an important role in food security and protein supply, providing 11.1% of the total global grain and 20.3% of the total human protein requirement [1]. The quality of wheat has a great impact on market value and consumer acceptance. Wheat quality is a complex and comprehensive concept [2], which mainly refers to the satisfaction and adaptability of grains to a specific use [3]. Broadly, wheat quality mainly includes nutritional and processing quality. Nutritional quality is related to the content of albumin and globulin, the composition of amino acids, especially the content of essential amino acids such as lysine, and the content of other nutrients [4,5,6]. Processing quality is further classified into primary and secondary processing quality. Primary processing quality, also referred to as milling quality, determines wheat’s suitability for the milling process. It mainly encompasses the physical characteristics of wheat grains (e.g., bulk density, 1000-grain weight, grain hardness, grain color) and their grinding properties (e.g., flour yield, flour whiteness, ash content). Secondary processing quality, alternatively termed food processing quality, refers to the processing adaptability and quality characteristics of wheat-flour-based products during the processing. The main evaluation indicators include sedimentation value, falling number, wet gluten content, gluten index, farinograph parameters, dough extensibility parameters, gelatinization parameters, and quality scoring of noodles, steamed buns, bread, and cakes [7].

Starch and protein are the two most abundant components in wheat grains, constituting approximately 65–70% and 7–18% of grain, respectively [1]. The content and characteristics of these two components during grain development play a critical role in determining the final yield and quality of wheat [8]. The concentration and composition of the grain protein determine the nutritional and end-use properties of the dough [9]. According to the difference in solubility, the protein in wheat grains is categorized into gliadin (soluble in 70–90% ethanol), glutenin (soluble in dilute acid and dilute alkali solution), albumin (soluble in water), and globulin (soluble in salt solution only). Among them, gliadins (molecular weight size of 30–80 kDa [10]) account for approximately 40–50% of the total protein content [11]. They are primarily classified into *α*/*β*-, *γ*-, and *ω*-types, and are responsible for imparting dough extensibility. In contrast, glutenins constitute about 50–60% of wheat grain proteins [11]. These proteins are divided into high-molecular-weight glutenin subunits (HMW-GSs, 70–140 kDa [10]) and low-molecular-weight glutenin subunits (LMW-GSs, 30–50 kDa [10]), which contribute to dough elasticity and strength. Gliadin and glutenin together constitute gluten protein and endow wheat flour with the unique properties required for the production of bread, other baked goods, pasta, and noodles, thereby primarily determining the processing quality of wheat. Albumin and globulin, which account for about 15% of wheat grain protein [12], are mainly related to the nutritional quality of wheat [13].

Based on chemical composition, wheat starch is divided into amylose and amylopectin, where amylose is a linear polysaccharide composed of D-glucose units linked by α-1,4-glycosidic bonds with few branches, while amylopectin shows a highly branched structure linked by α-1,6-D-glucosidic bonds [14]. Alterations in the amylose-to-amylopectin ratio affect the size and shape of starch granules and the interaction network of starch and protein in wheat endosperm [15]. Amylose and amylopectin content affect the dough and the quality of bread, while waxy wheat, characterized by low amylose content or near absence of amylose, can be utilized to enhance the nutritional quality of bread and other wheat-based products [16]. The granule size of wheat starch exhibits a bimodal distribution, which is widely acknowledged as the large A-type starch (d > 10 μm) and the small B-type starch (d ≤ 10 μm) according to the particle size, in which A-type starch accounts for 70–80% of the total starch, and B-type starch accounts for less than 10%, and there is also a mixture of A-type starch and B-type starch, called C-type starch [17]. The starch content and amylose content are higher in the A-type starch granule sample, while the B-type starch granule sample contains a higher proportion of protein and damaged starch. The proportion and content of starch granules with different sizes also play a crucial role in determining starch characteristics and overall wheat quality [18]. Compared with A-type starch granules, B-type starch granules are smaller but have a larger surface area, allowing them to be more evenly filled in the gluten network and form a denser dough structure [19].

In practical production, wheat quality is influenced by genotype, environmental variability, and their interaction [20]. Numerous environmental factors have been reported to affect wheat quality, including soil water availability, light intensity, soil characteristics, temperature, and elevated CO_2_ levels [21]. Water stress significantly impacts wheat grain quality, water deficiency during grain development leads to reduced starch biosynthesis, lower grain weight, decreased yield, and fewer B-type starch granules [22,23]. Another study found that increased moisture content correlates positively with protein content but negatively with starch content [24]. As an essential factor for photosynthesis [25], light availability directly influences crop photosynthetic efficiency, thereby modulating carbohydrate biosynthesis and protein accumulation as well as their distribution. Variations in light intensity and photoperiod during grain development alter the quantity of carbohydrates, proteins, and their structural properties, ultimately affecting processing quality. Typically, reduced light exposure suppresses carbohydrate synthesis while potentially enhancing protein accumulation [26,27,28,29]. Protein content varies significantly across soil types, following this trend: brown soil > fluvo-aquic soil > black soil. Soil texture (from sandy to clayey) exhibits an inverted U-shaped relationship with protein content, while organic matter enrichment typically enhances protein levels. Neutral soils are generally optimal for wheat cultivation [30,31]. Interestingly, certain wheat genotypes exhibit increased protein content but reduced yield under specific soil conditions, suggesting potential for breeding cultivars with both high yield and stable protein content [32]. Precision nitrogen management based on soil fertility and varietal yield demand characteristics at different growth stages can further optimize protein accumulation [33]. Moreover, rising atmospheric CO_2_ concentrations reduce nitrogen assimilation in wheat, leading to decreased concentrations of amino acids, proteins, and vitamins in grain. Concurrently, elevated CO_2_ reduces the content of essential dietary minerals (P, S, Zn, Fe, Cu, Mn, Mg) while increasing non-structural carbohydrates (such as starch and sugars), thereby compromising nutritional quality [34]. Temperature variability during grain formation and filling stages significantly impacts wheat grain quality, posing a global agricultural challenge [35]. Given the critical role of temperature in determining wheat quality, this review prioritizes it as a focal environmental factor. Numerous studies have shown that high and low temperatures limit the transfer of dry matter during wheat development, resulting in the reduction in wheat yield, grain appearance quality, content and proportion of each protein component, protein functional properties, and gluten strength. However, inconsistencies exist among different studies, which can be attributed to differences in affected intensity, the stage of action, the duration of exposure, and the used wheat genotype. This study investigates the effects of temperature on the composition of starch and protein in wheat grains at different developmental stages and the processing quality. In addition, the reasons for the influence of temperature on wheat quality traits are discussed.

## 2. Materials and Methods

This review examined articles that investigated the effects of daytime heat, nighttime heat, and low-temperature stress on wheat starch, protein, and processing quality. It summarized the current understanding of heat-induced impacts and highlighted inconsistencies in reported findings. The literature search was conducted using Web of Science, Scopus, PubMed, and library databases. The following keywords were used: “wheat quality”, “temperature stress”, “high temperature”, “low temperature”, “protein”, “starch”, “protein composition”, “starch composition”, “glutenin”, “gliadin” “amylose”, “amylopectin” “gluten strength”, “dough rheology”, “starch properties”, “baking quality”, “starch synthetase, and” “seed storage protein accumulation”.

## 3. Effect of Temperature on Wheat Quality

### 3.1. High Temperature

Since 1950, the global land average surface air temperature has risen by about 0.13 °C per decade [36]. It is estimated that the global average temperature will rise by about 0.35–0.70 °C by 2035 [35]. The increase in temperature caused by global warming has a particularly profound impact on wheat quality [37]. High temperature impacts the regulation of protein and starch content in wheat grains, thereby affecting the quality of wheat, especially the processing quality.

Studies have found that the increase in temperature positively affected the content of total protein, gliadin, and glutenin in wheat, but the ratio of glutenin to gliadin (Glu/Gli) decreased when the temperature exceeded 30 °C, which is detrimental to dough strength [38]. For example, Tanaka et al. [39] investigated changes in protein content and composition in Japanese commercial common wheat varieties (Norin 61) and heat-tolerant Sudanese common wheat varieties (Imam, Condor, Tagana, Bohaine, and VYT11) under high-temperature stress (38/18 °C, day/night) during maturity; they found that the total protein content and the proportion of total gliadin increased, but the proportion of total glutenin decreased, the total ratio of LMW-GSs decreased in all varieties, while the ratios of D-type LMW-GS, *α*-gliadin, and *ω*-gliadin increased. High temperatures appeared to predominantly affect the composition of polymer components (e.g., soluble and insoluble polymers), but not their synthesis. Zhao et al. [37] studied the effects of high-temperature stress (35 °C/22 °C day/night, 3 days) on the protein ratio at different grain-filling stages of two wheat varieties (Ningmai 13 and Zhenmai 12), and found that high temperature dramatically increased the proportion of total protein, gliadin (mainly ω-gliadin) and glutenin (mainly HMW-GS). In addition, the gliadin content increased more than the glutenin content due to temperature stress, resulting in a slight decrease in the glutenin/gliadin content under temperature stress. The most sensitive stage of grain main components to short-term temperature stress was 15–17 days post-anthesis (DPA), while temperature during the period of 31–33 DPA had the least effect on wheat quality. Kong et al. [40] studied the effects of temperature change on the protein components in winter wheat during the four key growth periods of re-greening, jointing, anthesis, and maturity; the results showed that winter wheat could partially offset the adverse effects of rising temperature on grain protein content, and the increasing temperatures prolonged the effective growth period, increased the total protein, gliadin and glutenin contents, and improved the bread processing quality, but reduced the albumin and globulin contents. Therefore, in a certain temperature range, warming is an effective way to improve the processing quality of winter wheat. Despite constituting only 10–12% of the total protein of the grain, HMW-GS plays an important role in determining the dough strength and accounts for 45 to 70% of the variation in bread-making quality [28,41]. Aono et al. [42] investigated the relationship between post-flowering temperature and storage protein properties of spring wheat (*Triticum aestivum* cv. Haruyokoi) under four temperature gradients below 30 °C, with a 2 °C difference between each gradient, and discovered that crude protein and SDS insoluble protein contents increased with increasing temperature, and HMW-GS 1Dx5 showed high accumulation at higher temperature.

Overall, when the temperature is lower than 30 °C, the protein content increases as the temperature rises. However, when the temperature exceeds 30 °C, the protein quality shows a decreasing trend with rising temperature. For example, Hernández-Espinosa et al. [43] analyzed the changes in protein content and dough strength in a set of 54 historical and modern spring bread wheat varieties (developed by CIMMYT and related breeding programs) under high-temperature conditions (35–39 °C) during the grain-filling stage. They found that the gluten strength decreased slightly under extremely high temperatures, but the protein content, gluten extensibility, and bread volume increased. Guzman et al. [44] examined the relationship between gluten strength and temperature in six durum wheat varieties (Mexicali C75, Yavaros C79, Altar C84, Atil C2000, Jupare C2001 and Cirno C2008). The results indicated that gluten strength reduction correlated with extreme heat (maximum temperature of 36 °C). Previous studies have also found that 30 °C appeared to be a critical threshold for dough strength: below 30 °C, higher temperatures enhanced dough strength, above 30 °C, further heating reduced dough strength [45]. However, the study by Fleitas et al. [46] found that the dough strength did not show a decreasing trend under high-temperature conditions (>30 °C). Therefore, the effect of high temperature on dough strength may be more genotype-dependent. Dough strength is influenced by protein content, Glu/Gli, and GMP [47]. Balla et al. [48] found that heat stress (35/20 °C, grain-filling stage) reduced the unextractable polymer protein content (UPP%) in five winter wheat varieties (Plainsman V, Fatima 2, Mv Mambó, Mv Mariska, and Bezosztaya 1). Chunduri et al. [49] found that under severe heat stress (32 °C/17 °C, day/night), glutenin decreased, gliadin increased, and the Glu/Gli ratio decreased.

The influence of high temperature on protein composition and content is ultimately reflected in the food processing quality indicators. For example, Mahdavi et al. [50] studied the quality changes under high-temperature conditions in 60 high-grade wheat lines (*Triticum aestivum* L., CIMCOG 1–60) and four native wheat varieties (Kouhdasht, Zagros, Karim, and Dehdasht); they found that the high temperature (maximum temperature > 35 °C) increased the gluten index, wet and dry gluten content, flour water absorption, ash content, lipid and bread volume of different spring wheat varieties, while the grain moisture content decreased. Meanwhile, the study also found that although certain parameters exhibited consistent response trends to elevated temperature across cultivars, the magnitude of these changes showed significant genotypic variation, indicating that the effect of high temperature on quality was affected by genotypes. Previous studies have also highlighted the impact of high temperatures on the processing quality of wheat. For example, high temperatures increased loaf volume, dough extensibility, development time, and mixing stability, but decreased dough tenacity and the tenacity/extensibility ratio [46]. High temperature (>35 °C) during certain stages of the grain-filling period reduces the flour mixing time and tolerance, consequently decreasing dough elasticity [51].

Rising temperatures change the starch composition in wheat, especially amylose and amylopectin content, which subsequently affects wheat processing quality [16]. Zhao et al. [52] investigated the effect of high temperature on starch content in two wheat cultivars (Yangmai 9 with low grain protein content and Xuzhou 26 with high grain protein content) during the grain-filling to maturity period under temperature regimes (34 °C/22 °C, 32 °C/24 °C, 26 °C/14 °C and 24 °C/16 °C). They found that high temperatures significantly reduced total starch and amylopectin content, while amylose content was only slightly affected. Kumari et al. [53] studied the effect of high temperatures (32 °C and 40 °C) on starch content in two wheat varieties (thermos-tolerant cv. HD3059 and thermos-susceptible cv. BT-Schomburgk) during the grain-filling period. They found that while amylopectin and total starch content decreased significantly with rising temperature, amylose content increased with temperature elevation, and the same finding was found in the study of Liu et al. [54]. Zhao et al. [37] studied the effects of high-temperature stress (35 °C/22 °C, day/night, 3 d) on starch at different stages of the grain-filling period in two wheat varieties (Ningmai 13 and Zhenmai 12); the results showed that high-temperature stress reduced the total starch and amylopectin content. At the same time, it was found that starch formation was most sensitive to temperature stress at 15–17 (DPA). However, Mahdavi et al. [50] studied 60 high-grade wheat lines (*Triticum aestivum* L., CIMCOG 1–60) and four native wheat varieties (Kouhdasht, Zagros, Karim, and Dehdasht); the results showed that the high temperature (maximum temperature > 30 °C) at the grain-filling stage increased amylopectin content and decreased amylose content. Liu et al. [55] (2023) investigated the effects of high-temperature stress (maximum temperature 35 °C) on starch content and related characteristics in waxy wheat Yangnuo 1 (YN1) and non-waxy wheat Yangmai15 (YM15) during different grain-filling stages (pre-, mid-, and post-filling). Their results showed that the amylose content significantly decreased in both varieties, the proportion of short chains in amylopectin increased, and the middle grain-filling period (16–20DAP) showed the most pronounced effects on amylopectin structure. Additionally, it was also found that high temperatures reduced starch crystallinity, gelatinization enthalpy, swelling potential, solubility, and light transmittance.

Environmental factors exert varying influences on final wheat quality at different wheat development stages. The physicochemical properties vary among different types of starch granules, and their particle size distribution also affects the rheological properties and processing characteristics of wheat dough. Liu et al. [56] compared the changes in the volume of A-type and B-type starch in two wheat cultivars (Yangmai 9 with weak gluten and Yangmai 12 with medium gluten) at 30 °C and 40 °C. They found that the volume of A-type starch granules decreased, while that of B-type starch granules increased significantly at 40 °C. Lu et al. [57] examined the effects of high temperature (38 °C, 5h/d, 7–10 DAP) on starch granule composition in high-yielding winter wheat cv. Zhengmai 366. They observed significant reductions in both A-type and B-type starch granule proportions under heat stress. Li et al. [58] investigated heat stress effects (37/28 °C, day/night, 5 DPA to maturity; control: 24/17 °C, day/night) on starch biosynthesis and degradation gene expression in winter wheat cv. Xindong 20. The results showed that high temperature significantly increased A-type granules while decreasing B-type granules, which is possibly due to the heat-induced destruction of amyloid bodies. In wheat, a well-documented negative correlation exists between protein and starch content. Elevated temperatures (>30 °C) typically enhance protein accumulation at the expense of starch content [59], a trade-off often accompanied by yield reduction. During the reproductive phase, photosynthesis was impaired when average temperatures exceeded 30 °C, which in turn led to premature ripening, reduced grain filling, and consequent yield loss [60].

The 1000-grain weight serves as an indirect indicator of starch content changes. Li et al. [61] studied the effects of high temperature (30 °C) at different stages (15/25/35 d) on wheat cv. Guizi 1. They found that high temperatures decreased the 1000-grain weight. Similar results were reported by Aono et al. [42], which may be attributed to heat-induced acceleration of maturity, which reduces starch accumulation with increasing protein content, thereby lowering grain weight. Liu et al. [55] showed that high temperature in the early stage of grain filling (6–10 DAP) significantly reduced both grain number and 1000-grain weight, whereas mid-filling heat (16–20 DAP) significantly decreased 1000-grain weight. In addition, high temperatures are accompanied by changes in other traits. For example, Rangan et al. [62] found that changes in grain width under high-temperature conditions (day/night, treatment: 38/20 °C; control: 20/18 °C) differed among three wheat genotypes. Banks and EGA Gregory exhibited significant reductions compared to the control, while Fang-60 showed statistically non-significant changes in grain width. Zhao et al. [37] studied the effects of high-temperature stress (35 °C/22 °C, day/night, 3 d) on the protein ratio of two wheat varieties (Ningmai 13 and Zhenmai 12) during various stages of grain filling, and found that high-temperature stress reduced the grain width.

Most studies have focused on daytime temperature effects on wheat quality, whereas research on the high nighttime temperature impacts remains limited. Current evidence suggests nighttime warming increases grain protein content but reduces starch accumulation. For example, Li et al. [63] conducted night warming treatment during grain-filling stage using the strong gluten cv. Xinong979 and the plain gluten cv. Tam107. They found that nighttime warming significantly increased grain protein content in both two varieties, accelerated UPP accumulation, enhanced dough micro-structure compactness, and improved flour processing quality. Giménez et al. [64] examined the higher nighttime temperature (4 °C increase compared to outdoor temperature) effects on the quality of two spring wheat varieties during the critical period (from third visible node to 10 DAP) and grain-filling period (10 DAP to physiological maturity). The results showed that the warmer nights shortened the time to anthesis while improving quality parameters, particularly grain protein concentration, gluten content, and dough baking strength. Impa et al. [65] identified a critical nighttime temperature threshold(23 °C) across 10 winter wheat varieties during flowering and grain-filling stages, beyond which a continuous decrease in quality but an increasing lipid accumulation were observed, and the different changes in starch and protein content were observed in tolerant genotype SY Monument and the sensitive genotype KS07077M-1. Previous studies have indicated that increasing lipid accumulation enhances free fatty acid oxidation, generating compounds responsible for undesirable aroma and taste that ultimately impair bread-making quality [66,67].

Other studies implementing nighttime warming treatments at different pre-anthesis stages have reported increased starch content and grain yield in wheat. For example, Fan et al. [68] conducted field experiments using four wheat cultivars with a passive nighttime warming method. They found that the nighttime warming during the tillering-jointing stage increased 1000-grain weight, sucrose, and starch content, consequently boosting wheat yields. However, the warming effect was more pronounced in semi-winter wheat varieties (Yannong 19, Annong 0711) than in spring wheat varieties (Yangmai 18, Shuimai 188), indicating genotype-dependent responses to high temperatures at night. In conclusion, moderate nighttime warming enhances protein accumulation. However, prolonged pre-flowering high-temperature, particularly when combined with elevated grain-filling temperatures, significantly impaird starch biosynthesis and consequently reduced grain yield. Interestingly, nighttime warming treatments from tillering to jointing stages may benefit grain starch accumulation.

**Figure 1:**
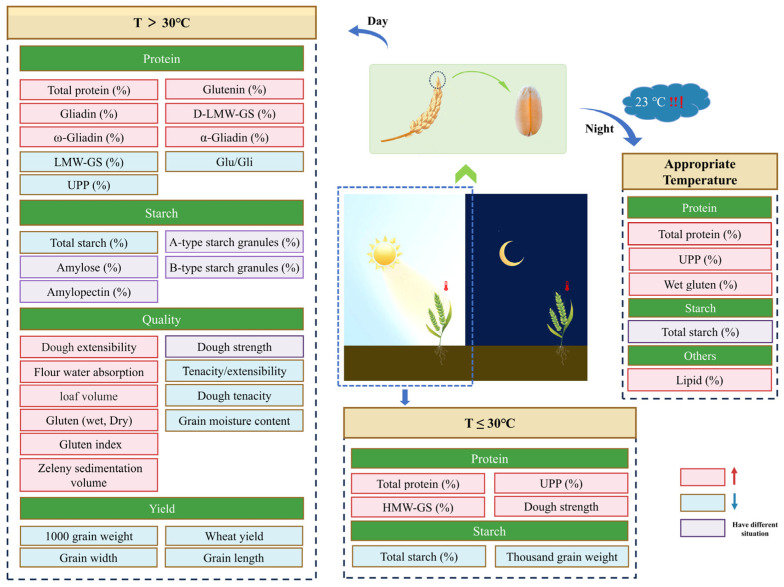
Effects of high day/night temperatures on protein, starch, and other wheat qualities.

In summary, increasing daytime temperatures within a certain range (<30 °C) during wheat growth generally improves wheat quality. However, temperatures exceeding 30 °C typically reduce protein quality, ultimately compromising wheat quality. Conversely, controlled nighttime warming enhances wheat quality by increasing protein content and UPP proportion. This improvement, however, comes at the expense of starch synthesis and yield potential. Moreover, as reported by Impa et al. [65], 23 °C may be a beneficial nighttime temperature threshold (Figure 1).

### 3.2. Low Temperature

The rising global average temperature in recent decades has led most studies to focus on the heat stress effect on grain quality [69], while low-temperature impacts remain understudied. Similar to heat stress, low-temperature effects on wheat quality depend on multiple factors: frequency, intensity, duration, and the specific growth stage when low-temperature events occur.

Previous studies indicated that winter wheat is most vulnerable to low-temperature stress during jointing and booting stages when optimal average temperatures range between 9.3 and 11.9 °C [70]. Liu et al. [69] conducted a two-year temperature control experiment with two winter wheat varieties (Yangmai16 and Xumai30) under varying low-temperature regimes (T1: T_min_/T_avg_/T_max_, 6/11/16 °C, CK; T2: 2/3/8 °C, T3: −4 /1/6 °C; T4: −6/−1/4 °C). They found that low temperature generally enhanced protein-related quality parameters, including increased total protein, gliadin, albumin, globulin content, higher wet and dry gluten concentration, and improved SDS sedimentation, with these improvements increasing as low-temperature exposure duration and intensity increased. However, glutenin content showed stage-dependent variation. Furthermore, the results indicated that the intensity of low-temperature stress had a more significant impact on wheat grain quality than its duration. Additionally, wheat grain quality at the booting stage was more cold-sensitive than at the jointing stage. Similar conclusions were reported by Shi et al. [71] and Zhang et al. [72]. However, Zhang et al. [72] noted that although low temperature increased protein concentration at the jointing and booting stages, it actually reduced the accumulation (mg·grain^−1^) of total protein and various components (albumin, globulin, gliadin, glutenin).

The optimal temperature range for wheat grain filling is 20–22 °C [73]. Labuschagne et al. [21] studied protein changes in bread wheat (cvs. Kariega and SST86) and durum wheat (cv. Oranje) under extremely low temperatures (−5.5 °C, 3 h) during the grain-filling period across two growing seasons. The results showed that low temperatures significantly reduced SDS sedimentation values but increased the protein content across all varieties. The increase in protein content was mainly due to an elevation in soluble (gliadin) protein [74]. LMW-GS are classified as B-type, C-type, and D-type. B-type LMW-GS is the main group of LMW-GS that can act as glutenin polymer chain extenders, while C-type and D-type LMW-GS are terminators of glutenin polymers [75]. Koga et al. [76] investigated low- to medium-temperature effects (T1: 13/10 °C, day/night; T2: 18/15 °C, day/night; T3: 23/20 °C, day/night) on gluten quality in two spring wheat varieties (Bjarne and Cadenza) during grain filling. The results revealed that although both varieties possessed the superior subunit 1Dx5+1Dy10, the low temperatures significantly reduced the UPP content in Cadenza, while Bjarne maintained a high UPP proportion across all temperatures, indicating that glutenin polymer assembly in Bjarne was less sensitive to temperature changes compared to Cadenza, which suggested that glutenin polymer assembly is mainly determined by genotype. Meanwhile, low temperature also decreased the total protein, ω-gliadin, and D-type LMW-GS proportions, whereas α- and γ-gliadin and B-type LMW-GS proportions increased as temperature decreased. These compositional changes affect glutenin polymer formation. Koga et al. [77] subsequently investigated the effect of the aforementioned three temperatures on gluten function and gluten protein composition in four spring wheat varieties (Avle, Berserk, Bjarne and Zebra) during the grain-filling period. The study revealed consistent patterns of gluten protein fraction variation across all varieties, with the HMW-GS proportion remaining stable among three temperature treatments. Notably, sustained low temperatures down to 13 °C showed no adverse effect on maximum resistance to extension (R_max_). The Berserk variety maintained a consistently high R_max_ value regardless of temperature, further indicating the genotypic influence on low-temperature responses in gluten quality. These findings align with the previous studies, in which both low temperatures (9–18 °C) and cool, wet weather during the wheat-grain-filling period reduced gluten strength [78].

Some studies have shown that low temperatures reduce total starch and amylose content and the amylose/amylopectin ratio in wheat grains. Zeng et al. [79] examined the impact of extremely low temperatures (minimum −40 °C) on the starch content in two winter wheat varieties, cold-resistant Dongnongdongmai 1 (survives at −30 °C) and weakly cold-resistant Jimai 22 (survives only above −10 °C). The results showed that low temperature decreased concentrations of major soluble sugars (sucrose and fructose) and reduced starch content. Liu et al. [69] conducted a two-year temperature-controlled experiment in an artificial climate chamber using two winter wheat varieties (Yangmai16 and Xumai30). They found that low temperatures (−2/−4/−6 °C) negatively affected starch concentration. Additionally, amylopectin content was more sensitive to low temperatures than amylose content, with a higher degree of decline. Zhang et al. [80] observed reduced starch and dry matter accumulation in cold-tolerant Yannong 19 and cold-sensitive Yangmai 18 when exposed to nocturnal low temperature (−2/0/2 °C from 19:00 to 07:00, and 5 °C from 07:00 to 19:00; control: without cold stress). Further investigation by Zhang et al. [81] on Wanmai 52 and Yannong 19 at booting stage under varying low temperatures (T1: 2/5 °C, 12/12 h, day/night; T2: 0/5 °C, 12/12 h, day/night; T3: −2/5 °C, 12/12 h, day/night; T4: CK, no treatment) confirmed significant reductions in both amylose and amylopectin content). These starch changes exhibit genotypic dependence, as demonstrated by Labuschagne et al. [21] showing differential starch content responses among bread wheat Kariega, SST86, durum wheat Oranje, and soft biscuit wheat snack under −5.5 °C exposure. In addition, low temperatures (−2/−4/−6 °C) impaired grain appearance quality, decreasing grain length and width while increasing length–width ratio [69].

**Figure 2:**
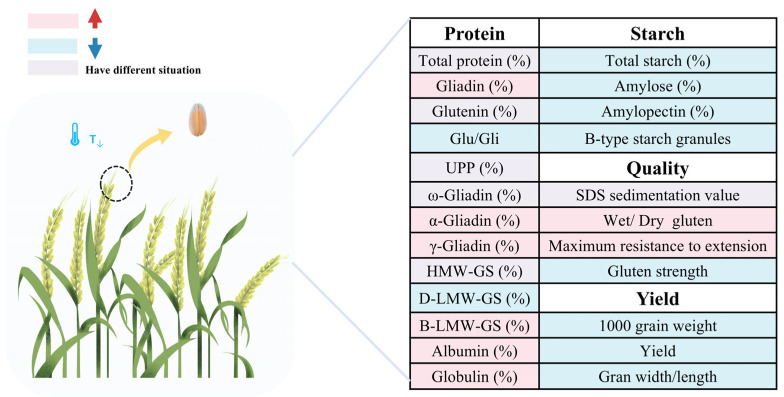
Effects of low temperatures on protein, starch, and other wheat qualities.

In summary, low temperatures predominantly influence wheat quality during grain filling, jointing, and booting stages, with booting being the most sensitive. Low temperatures reduced starch content, while the protein content varied depending on the treated temperatures and varieties (Figure 2) (Table 1).

## 4. Mechanism Underlying Temperature-Driven Quality Changes in Wheat Grains

The impact of temperature on wheat quality varies depending on the developmental stage and ambient temperature conditions. The accumulation rates of key grain components (proteins and starches) exhibit pronounced temperature dependence throughout its growth. Elevated temperatures typically reduce kernel starch content due to a shortened maturation period, adversely affecting starch-related quality traits such as thousand kernel weight while simultaneously increasing protein content [1]. In general, low temperatures extend the flowering to maturity period, whereas high temperatures substantially shorten this critical phase [42]. Winter warming may accelerate flowering initiation, while cooler average temperatures during vegetative growth prolong tillering, spike differentiation, and grain-filling durations, thereby enhancing leaf area index and biomass accumulation [82]. The grain-filling rate plays a pivotal role in dry matter accumulation and ultimately wheat quality [83]. However, the temperature effects on grain-filling rate remain debated. Some studies found that both short-term high (35/22 °C) and low temperatures (18/8 °C) reduced grain-filling rates. Under low-temperature stress, prolonged filling durations failed to offset the adverse effects of reduced filling rates on grain weight [37]. Conversely, other research demonstrates that elevated growth temperatures (20–28 °C) increase grain-filling rates in a variety of spring and winter wheat cultivars at the maturity stage, partially offsetting the impacts of shortened filling periods [84,85]. These discrepancies likely stem from variations in temperature regimes, developing timing of stress exposure, or both factors. Vernalization requirements further complicate this relationship, as climate warming may prevent winter wheat from experiencing sufficient chilling (either in intensity or duration) for proper flowering initiation [86]. He et al. [87] found that temperature increases accelerate phenology markedly, with each 1 °C rise advancing flowering by approximately 10 days and shortening later developmental phases by 4.1 days /℃ in the flowering period of wheat. Pre-flowering warming may accelerate the transition from jointing to heading stages, consequently elevating the risk of frost damage and late-spring cold injury. Furthermore, extended exposure to low temperatures can induce chilling stress in crops, negatively impacting both yield potential and grain quality [88].

Under high-temperature conditions, elevated storage protein content may be attributed to the following factors. Farooq et al. [73] proposed that heat-stress-induced protein accumulation serves as a compensatory mechanism for starch content reduction, while Wang et al. [89] attributed this phenomenon to altered energy allocation under heat stress. Under high-temperature conditions (37 °C), plants preferentially direct energy toward stress response rather than metabolism process, thereby reallocating photosynthetic assimilates from routine metabolism to thermal protection and reserve deposition. This shift ultimately enhances storage protein accumulation and stabilization of the dry matter filling rate in grains. Alternative explanations for elevated protein content were proposed, including the source–sink relationship (leaf-to-grain translocation), carbon–nitrogen allocation dynamics during grain development and metabolism in leaf and grain [90], as well as the temporal coordination of carbon and nitrogen assimilation in endosperm cells [91]. Additionally, certain studies have indicated that the rate of carbohydrate uptake is usually lower than that of nitrogen uptake under increasing temperatures, which favors the accumulation of nitrogenous substances and ultimately leads to a higher rate of protein synthesis in grains [92]. In addition to the above three points, Kong et al. [40] identified an additional mechanism whereby warming reduces fertile spikelet number in winter wheat, concentrating available nitrogen into fewer developing kernels and consequently elevating their protein content. Enhanced photosynthetic performance under moderate heat stress, manifested through increased leaf area index, biomass production, and nitrogen uptake, further contributes to protein accumulation [93,94,95,96]. In contrast to the heat-induced increase in storage protein content, the relative content of albumin and globulin, which are related to nutritional quality, exhibited a declining trend as temperature increased [40]. Albumin and globulin contain more lysine, whereas gliadin and glutenin contain more glutamine. The increasing of gliadin and glutenin relative content was beneficial to the processing quality, but not to its nutritional quality. However, some studies have found that the temperature increase in a certain range combined with no-tillage may increase the protein content, which is conducive to balancing the processing and nutritional quality of winter wheat [1].

Under high-temperature conditions, the decreasing starch content may be due to several factors. Starch accumulation in wheat grains is derived from two sources: post-anthesis photosynthesis in leaves, ears, and other green tissues, and remobilization of pre-anthesis non-structural carbohydrates stored in vegetative organs such as sheaths and leaves [97]. Gebbing et al. [98] found that pre-anthesis non-structural carbohydrates contribute 8–27% to grain development, with thermal stress impairing both carbon fixation efficiency and translocation capacity, ultimately reducing starch accumulation in grains. The bimodal distribution and the basic shape of the mature A- and B-type starch granules remain largely unaffected by high temperatures, suggesting strong genetic control over these traits [99]. However, the grain-filling period is shortened under high-temperature conditions, and the intensity and duration of stress accumulated during this stage are likely to negatively impact the development of B-type granules. High temperatures may severely disrupt amyloplasts, leading to a significant reduction in B-type granules and allowing larger A-type granules to dominate the starchy endosperm. Moreover, the degree of starch accumulation is determined by the grain volume, which may be influenced by external tissue, carpel weight, endosperm cell size, and other factors [100]. Based on Clifton’s study [101], the reduction in starch content under higher temperature conditions (38 °C) might be mitigated by a low nighttime temperature (5 °C). This reduction may be attributed to the significantly lower carbon loss during nighttime respiration under low nighttime temperatures [102], as well as the mitigation of daytime heat stress and the respiration advantages observed in tolerant genotypes [103]. However, current studies have shown that grain yield is positively correlated with starch content but negatively correlated with protein content [104]. The negative correlation between grain yield and protein content is a major problem in global wheat research [19]. It was found that this negative correlation can be interrupted by an elevated temperature, which increases the protein content and yield of winter wheat. This may be attributed to the extension of the effective reproduction period, and the increase in available light energy and accumulated temperature, which enhance the net photosynthetic rate of the winter wheat population after flowering, ensure post-anthesis material production, and contribute to winter wheat yield increases in North China [1]. Therefore, investigating the impact of environmental factors on quality may provide insights into addressing the dilemma of the negative correlation between yield and quality.

The alterations in protein and starch under low-temperature conditions are attributed to a multitude of factors. Pre-flowering low-temperature stress inhibits the synthesis of non-structural carbohydrates and nitrogen compounds in the nutrient organs, thereby affecting mineral uptake and distribution in wheat [105,106,107]. Post-flowering low temperatures may reduce grain-filling rate [108], slow the transport of proteins and total soluble sugars from stem to seed, and ultimately degrade wheat quality [109,110]. Low temperatures during early jointing can damage wheat’s functional leaves, inhibit organic matter synthesis, alter the source–sink relationship, and disrupt organic nutrient supplementation during endosperm cell proliferation and enrichment. These changes ultimately impair later-stage grain development, affecting both grain composition and final morphology [111]. Downregulated differentially expressed proteins (DEPs) involved in starch and sucrose metabolism, such as sucrose phosphate synthase (SPS), glucose-1-phosphate adenylyltransferase (glgC) and β-fructofuranosidase (FFase), ultimately hinder starch synthesis. However, under low-temperature conditions, the upregulation of seed storage proteins (SSPs) plays a positive role in mitigating low-temperature stress and its subsequent damage [81]. The low-temperature-induced reduction in starch content may also result from impaired photosynthesis, which reduces carbon accumulation [69]. In addition, 0 °C serves as the threshold temperature for frost damage. Chilling injury occurs above 0 °C, delaying crop flowering, causing direct tissue damage, or reducing plant viability. In contrast, frost damage occurs below 0 °C. When air temperature approaches below freezing, plant tissues drop below the ambient temperature, leading to ice crystal formation on leaves, stems, and flowers. These ice crystals puncture cell membranes, causing physical damage, while intracellular freezing induces dehydration, disrupting photosynthesize. Once physical damage occurs, brown to yellow necrosis develops, resulting in partial or complete plant death [112].

Elevated temperatures may reduce enzyme activity, leading to alterations in wheat quality. The key enzymes controlling protein synthesis in wheat include glutamine synthetase (GS), glutamate synthase (GOGAT), glutamic-pyruvic transaminase (GPT), and different isoforms of the protein disulfide isomerase (PDI) family. Among them, GS is closely linked to wheat grain quality. GS, with ATP participation, catalyzes nitrogen assimilates into glutamine [113]. One study demonstrated that glutamine concentrations play a major role in gluten biosynthesis during grain maturity [114]. GOGAT works synergistically with GS to maintain the glutamine/glutamate metabolism cycle, while GPT regulates nitrogen transfer from glutamate (its main carrier) to other protein-forming amino acids [115]. PDI is involved in gluten macropolymer (GMP) formation and ensures proper folding and accumulation of stored proteins in the endosperm [116,117]. Elevated temperatures impair the activity of storage protein biosynthesis-related enzymes, consequently compromising wheat quality. Aono et al. [42] demonstrated that while moderate heating (<30 °C) elevated protein content, it suppressed protein disulfide isomerase (PDI) activity. Notably, this suppression did not hinder disulfide (S-S) bond formation, suggesting that mild heat-induced dysfunction of intramolecular disulfide-bonding enzymes promotes non-native protein folding and subsequent nonspecific storage protein aggregation. Currently, limited information exists on high-temperature effects on wheat grain protein synthase. Although many studies have been carried out on the influence of environmental factors on storage protein accumulation, the effect of temperature on protein synthase during grain filling is still unclear compared with starch biosynthesis, and only a few studies have addressed the effect of temperature on protein synthesis-related enzyme activity. For example, Chen et al. [118] reported that temperatures exceeding 30 °C inhibited GS activity in a duration-dependent manner. Short-term heat exposure (≤3 days) had no significant impact, whereas extended treatment (>3 days) markedly reduced GS activity. Notably, enzymatic function was restored upon cessation of thermal stress. Lu et al. [115] found that the effect of high temperature (>30 °C) on GPT activity varied by variety. Zhao et al. [119] showed that high temperatures (>30 °C) reduced the activity of GPT and GS, with grain protein content negatively correlating with the activities of key enzymes (GPT, GS). Notably, GPT activity strongly correlated (*p* < 0.01) with protein yield, while GS activity positively correlated with protein content. One study noted upregulation of Pre a/β gliadin, γ-gliadin, and LMW metship7 genes under daytime and day–night high temperatures, but downregulation of Glu trip prt 3 (a glutenin synthesis precursor gene) [49], accompanied by reduced glutenin content but increased gliadin content and elevated total storage protein. Low-temperature stress also has an effect on enzyme activity. Majláth et al. [120] found that low temperature reduced GS activity during nitrogen compound assimilation, which may ultimately lead to decreased storage protein content. However, most of these studies did not directly link enzyme activity changes to shifts in protein content and composition.

Compared with studies on the effects of elevated temperature on enzymatic activities in storage protein biosynthesis pathways, more research attention has focused on temperature impacts on key enzymes involved in starch biosynthesis. During wheat grain development, high temperature significantly affects starch-related enzyme activities, which critically influence starch biosynthesis. The starch in wheat grains is mainly synthesized from sucrose through a series of enzymes [121]. Starch biosynthesis constitutes a complex metabolic pathway involving several key enzymes, including sucrose synthase (Susy), ADP-glucose pyrophosphorylase (AGPase), granule-bound starch synthase (GBSS), Soluble starch synthase (SSS), starch branching enzyme (SBE), and debranching enzyme (DBE), which coordinately regulate the intricate starch production process [122]. Among these enzymes, SuSy is considered a key rate-limiting factor in starch synthesis. AGPase catalyzes the formation of adenosine diphosphate glucose (ADPG), the primary substrate for starch biosynthesis. GBSS mediates the conversion of ADPG to amylose, while SSS, SBE, and DBE collectively regulate amylopectin production [58].

Current research on high-temperature effects on starch can be categorized into three primary treatment types based on temporal patterns: daytime high temperature, nighttime high temperature, and day–night high temperature. Among these, daytime high temperature has received the most extensive research attention. Lu et al. [123] found that high day temperatures (>30 °C) reduced the activity of starch synthesis-related enzymes (Susy, SSS, AGpase, SBE, GBSS) and decreased starch content. Li et al. [58] showed that high day temperatures (>30 °C) upregulated α-amylase enzyme activity, enhanced starch degradation, and accelerated starch hydrolysis relative to starch synthase activity, resulting in reduced starch accumulation. Harris et al. [124] demonstrated that daytime high temperatures (<30 °C) significantly decreased AGPase and SSS activities (with SSS being the most temperature-sensitive enzyme), while having minimal effects on SBE, GBSS, and α-amylase. The differential responses among starch-degrading enzymes may reflect genotype-specific and/or temperature-dependent regulation. The observed reduction in biomass accumulation appears primarily attributable to heat-induced suppression of AGPase and SSS activities, which consequently impairs sucrose uptake, alters carbon partitioning, and ultimately diminishes starch deposition. High temperature altered the timing of starch biosynthesis and induced early peaks in gene expression during this process. Under high-temperature conditions, the transcriptional profiles of some starch synthesis-related genes AGPase, SSS, and SBE showed different expression levels at different growth stages, especially at 7 and 14 DPA [58]. Lu et al. [123] further demonstrated that high day temperatures (>30 °C) downregulated most starch biosynthesis genes (AGPL1, AGPL2, AGPS1-a, AGPS1-b, AGPS2, SSI, SSIIa, SSIIb, SSIIc, SSIIIa, SSIIIb, SSIV, GBSSI, GBSSII, BEI, BEIIa, BEIIb, BEIII, ISA1, PUL, PHOL, and PHOH) with the exception of ISA2, leading to reduced amylose, amylopectin, and total starch content. Chunduri et al. [49] investigated the effects of both daytime (35/17 °C) and day–night (35/24 °C) high temperatures on starch synthesis-related genes; they found that high daytime temperatures induced earlier expression of the starch transporter gene BT1, while high day–night temperatures resulted in lower expression levels. Meanwhile, the study also revealed decreased expression of other starch synthesis-related genes (AGPL1, AGPS1, GBSSI, GBSSII, SS1, SS2, SS3, ISA1, ISA2) and transcription factors (TaRSR1, OsbZIP58) under both temperature regimes. Notably, BMY (β-amylase) activity also decreased under both temperature conditions, indicating diminished energy supplementation through the metabolic pathway.

For high night temperatures, Impa et al. [65] investigated the gene expression levels of starch synthesis-related enzymes and found that post-flowering high night temperatures (27 °C) increased transcription levels of SUSI, ISAI, ISAII, and ISAIII, while decreasing expression of AGPLS, AGPSS, SBEI, GBSSI, and GBSII. Concurrent upregulation of the starch-degrading enzyme BMY further limited starch synthesis and accumulation in developing wheat grains under nocturnal heat stress. High-temperature stress appears to modulate gene expression through upstream regulatory elements. Arenas-M et al. [59] identified a novel NAC-type TF (TRITD5Bv1G096580) through regulatory network analysis. This TF targets glycogen and starch biosynthesis and showed significant heat-induced downregulation, participating in starch metabolism response to thermal stress in durum wheat. The impairment of starch biosynthesis enzyme activity directly correlates with downregulated expression of starch biosynthesis-related genes. Under heat stress, starch content decreases due to suppressed activity of key starch synthase and downregulated starch synthase genes [123]. In developing wheat endosperm, some starch may be broken down by α-amylase due to heat stress [125], and the starch content decreases when the decomposition rate exceeds the synthesis rate.

Low-temperature stress similarly disrupts starch synthesis-related enzyme activity. Zhang et al. [80] observed that short-term low-temperature stress during grain filling depressed AGPase, SSS, GBSS, and SBE activities, with starch accumulation rate and starch content declining as temperature decreased. Phosphorylase (PHO1) serves as a multifunctional enzyme critical for the initial starch biosynthesis stage, especially in the elongation of starch primers and the initiation of starch synthesis [126,127], while disproportionating enzyme (DPE1) remodel amylose and amylopectin molecules in cereal crops [128]. Zhang et al. [129] found that low-temperature stress at the booting stage downregulated the activities of key enzymes (PHO1 and DPE1) in the early stage of starch synthesis and the expression of enzyme-related genes such as AGPase, GBSSI, SSSI, SSSII, and PHO1.

**Figure 3:**
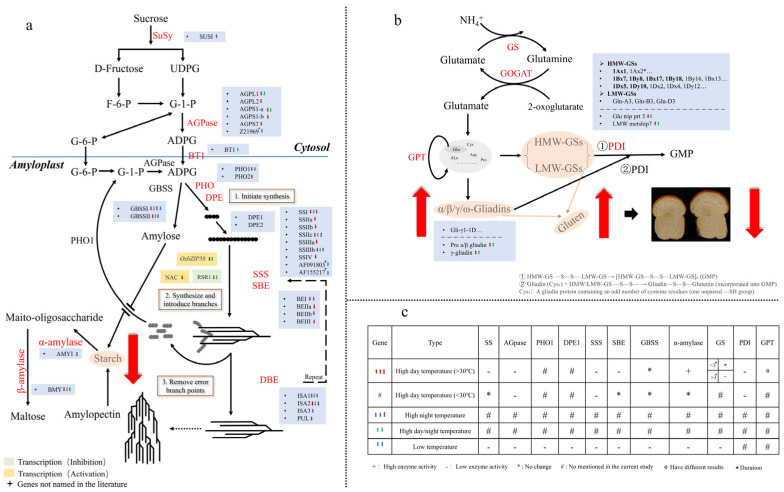
This diagram illustrates the temperature response mechanism of enzymes and genes involved in starch and storage protein synthesis. (**a**) Biosynthesis pathway of endosperm starch in wheat grains; (**b**) synthesis process of storage protein; (**c**) effect of high temperature on enzyme activity during both starch and storage protein synthesis process. The large red arrow in (**a**,**b**) represents the common effect of high and low-temperature stress on protein and starch metabolism; The small arrows in (**a**,**b**) represent gene upregulation or downregulation, different colors correspond to the different temperature conditions, which are listed in (**c**), with the arrow pointing up/down indicating positive/negative effects.

Overall, enzyme activity and gene expression were synergistically downregulated under temperature stress. Both high and low temperatures inhibited starch synthase activity and gene expression, while activating degrading enzymes such as α-amylase, resulting in reduced accumulation of stored substances (particularly starch). For storage proteins, while high temperatures (>30 °C) decreased the activity of protein-related enzymes, total protein content may increase due to nitrogen metabolism repartitioning (compensatory increase in gliadin) (Figure 3). Current research has two significant gaps: insufficient investigation of transcription factor regulatory networks (e.g., NAC type) in temperature stress response, and systematic studies on temperature effects (especially low temperatures) on glutenin/gliadin components.

## 5. Conclusions and Future Perspectives

High and low-temperature stresses significantly impact wheat grain appearance, protein and starch content, and compositional ratios, ultimately influencing the final processing quality. Overall, both cold and heat treatments consistently increase the total protein content while decreasing the starch content. Current research primarily focuses on the effect of temperature on two wheat species *Triticum aestivum* L. (bread wheat) and *Triticum durum* L. (durum wheat), with bread wheat receiving more extensive study. Guzmán et al. [44] demonstrated that under high-temperature conditions, durum wheat typically yields smaller loaf volume than bread wheat, though certain durum varieties can approach or even match the performance of superior bread wheat varieties under specific environments, such as severe heat stress. Wheat varieties are commonly classified by growing season as winter or spring types, previous studies found that global warming has a greater impact on winter wheat yield than spring wheat [130]. Temperature effects vary by timing and wheat type: daytime heat increases protein content but decreases starch in both winter and spring wheat, while moderate nighttime warming benefits starch accumulation, particularly in semi-winter varieties [68]. On the other hand, due to the importance of low vernalization temperature to winter wheat, the effect of winter temperature increases on winter wheat quality may be greater than that of spring wheat. Under low-temperature conditions, the decrease in temperature had an adverse effect on the starch content of both winter and spring wheat varieties. However, the changes in protein content were different, and low temperatures appeared to adversely affect the protein content of spring wheat [76]. Current research faces limitations in comparability due to variations in experimental designs (temperature stages, levels, small sample sizes), cultivars, and experimental conditions (inter-annual variations, agronomic practices). These methodological differences highlight the need for future studies employing standardized, multi-environment trials with larger sample sizes to improve data reliability and cross-study comparisons.

Although numerous studies have investigated temperature responses in wheat, most have focused on phenotypic analyses and changes in protein- and starch-related genes under temperature variations. Research on the identification of high and low-temperature tolerance genes in wheat remains limited. Compared to functional genes associated with major traits like disease resistance, quality, and yield, progress in identifying temperature tolerance genes has been relatively slow. The main quantitative trait loci (QTLs) directly linked to high-temperature tolerance have not yet been identified, and the mechanism of some discovered high-temperature tolerance genes remain incompletely understood [131]. To date, a number of high-temperature tolerance genes in wheat have been identified, including *EF-TU* [132], *TaGCN5* [133], *TaNAC2L* [134], *TaMyb80* [135], *TabZIP28* [136], *TabZIP60* [136], *HvSUT1* [137], *TaFER-5B* [138], *TaOEP16-2-5B* [139], *TaPEPKR2* [140], *TaGASR1* [141], *TaHsfC2a* [142], *TaHsfB2d* [143], *TaHsfA2-1* [144], *TaHsfA2-11* [145], *TaHsfA6f* [146], *TaHsfA1* [147], *TaFBA1* [148], *TaHSP23.9* [149], *TaOPR3* [150], *TaSINA* [151], *TaMBF1c* [152] and *TaHAG1* [153], *TaBZR2-3B* [154], *TaSG-D1^E286K^* [155] and *TaGRAS34-5A* [156]. The majority of these genes encode transcription factors that enhance high-temperature tolerance by regulating the expression of heat shock protein genes. Regarding low-temperature tolerance, several important genetic loci have been identified, including *Fr-1* [157], *Fr-2* [157], *QFr.jic-5D* [158], *Vrn-D1* [159], and *QFT.ahau-7B.2* (candidate gene: *TaRPM1-7BL*) [160]. Additionally, several key cold-tolerance-related genes such as *TaDi19A* [161], *TaSnRK1α* [162], and *TaPAP6L* [162] have been characterized. However, the specific candidate genes for most cold tolerance loci still require further clarification. Significant gaps remain in our understanding of both heat and cold tolerance mechanisms in wheat, highlighting the need for continued research on gene discovery and functional analysis.

Given the increasing variability of global climatic conditions, wheat varieties with only a single stress tolerance trait are no longer able to cope with complex environmental challenges. There is an urgent need to develop new wheat varieties with integrated environmental adaptations, which should simultaneously possess the following: (a) Multiple stress tolerance (including drought and heat resistance). (b) Broad vernalization temperature adaptability to address the impact of rising global temperatures on winter wheat vernalization requirements. (c) Elevated CO_2_ utilization efficiency. It has been found that elevated CO_2_ can partially mitigate the negative effects of heat stress on yield within a certain range, but this effect will be weakened or even disappear under prolonged extreme high temperatures [163], which is also accompanied by undesirable reductions in glutenin and gliadin content that impair quality [164]. (d) Balanced yield and quality characterization, especially maintaining an optimal Glu/Gli ratio. The development of such multi-environmentally adapted varieties will be a key strategy to address the challenges of food security in the face of climate change.

**Figure 4:**
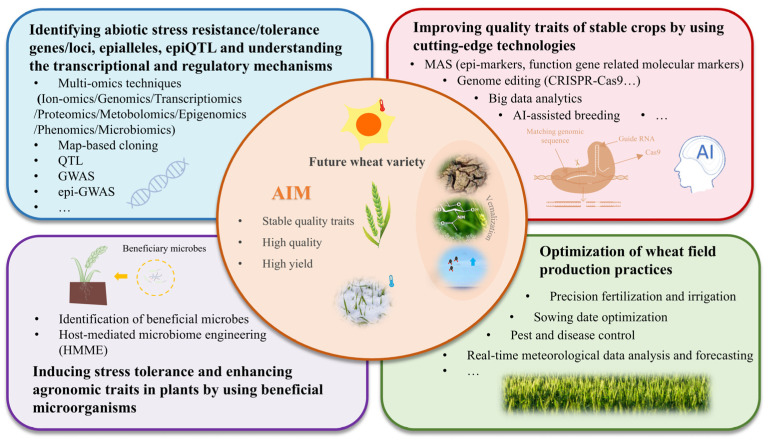
Strategies for breeding high-quality wheat under changing environmental conditions.

To develop high-quality, high-yield wheat varieties with stable traits, future research should integrate multidisciplinary approaches (Figure 4). (a) First, constructing genotype-protein/starch response maps could elucidate the regulatory mechanisms of protein and starch metabolism across different genotypes under environmental changes (e.g., temperature, drought). This should be coupled with multi-omics analyses, map-based cloning, QTL mapping, GWAS (genome-wide association study), and epi-GWAS approaches to identify the loci/genes, epialleles, epiQTL, and mechanisms associated with abiotic stress tolerance [165,166]. (b) Second, by using the corresponding molecular markers and epi-markers, molecular marker-assisted selection (MAS) [167] could enable early screening of wheat varieties with superior environmental adaptability and quality traits. Advanced gene editing technologies like CRISPR/Cas9 [168,169] could further allow precise modification of key genes or upstream transcription factors related to heat tolerance, drought resistance, and cold resistance. Moreover, the integration of deep learning, big data analytics, and AI-assisted breeding, screening, and prediction platforms could enhance the efficiency of desired trait screening and predictive breeding [170]. (c) Third, microbiome engineering approaches could be employed to harness beneficial plant–microbe interactions that enhance wheat’s abiotic stress resilience [171]. (d) Finally, implementing precision agricultural techniques, including real-time irrigation/climate monitoring, optimized planting schedules [172], and targeted fertilization, can counteract environmental stress-induced yield and quality losses in wheat.

## Figures and Tables

**Table 1:** Effects of low temperatures on protein, starch, and quality of wheat.

Treatment Stage	Temperature	Variety	Change ^1^	Reference
**Controlled Phytotron Glasshouse**				
**Starch**—Total starch content (%)				
Jointing period, Booting period	Four temperature levels: T_min_/T_avg_/T_max_, 6/11/16 °C (CK), 2/3/8 °C, −4 /1/6 °C, −6/−1/4 °C Three treatment durations: 2/4/6 days	Winter wheat (Yangmai16, Xumai30)	Decrease	[69]
Jointing period, Booting period	Three temperature levels: day/night, 6/16 °C (CK), 0/10 °C, −6/4 °C Two treatment durations: 3/6 days	Winter wheat (Yangmai16, Xumai30)	Decrease	[72]
Booting period	Four temperature levels: −2, 0 or 2 °C from 19:00 to 07:00, and 5 °C from 07:00 to 19:00, without cold stress (CK)	Semi-winter (Yannong 19), spring wheat (Yangmai 18)	Decrease	[80]
Filling period	18/8 °C (day/night), 3 d	Winter wheat (Ningmai 13, Zhenmai 12)	Decrease	[37]
Filling period	−5.5 °C, 3 h	Spring wheat (Kariega)	Decrease	[21]
**Starch**—Amylose (%)				
Jointing period, Booting period	Three temperature levels: day/night, 6/16 °C (CK), 0/10 °C, −6/4 °C Two treatment durations: 3/6 days	Winter wheat (Yangmai16, Xumai30)	Decrease	[72]
Jointing period, Booting period	Four temperature levels: T_min_/T_avg_/T_max_, 6/11/16 °C (CK), 2/3/8 °C, −4 /1/6 °C, −6/−1/4 °C Three treatment durations: 2/4/6 days	Winter wheat (Yangmai16, Xumai30)	Decrease	[69]
Booting period	Four temperature levels: day/night, 12/12 h, 2/5 °C, 0/5 °C, −2/5 °C, without Low temperature treatment (CK)	Winter wheat (Wanmai 52), semi-winter (Yannong 19)	Decrease	[81]
**Starch**—Amylopectin (%)				
Jointing period, Booting period	Four temperature levels: T_min_/T_avg_/T_max_, 6/11/16 °C (CK), 2/3/8 °C, −4 /1/6 °C, −6/−1/4 °C Three treatment durations: 2/4/6 days	Winter wheat (Yangmai16, Xumai30)	Decrease	[69]
Jointing period, Booting period	Three temperature levels: day/night, 6/16 °C (CK), 0/10 °C, −6/4 °C Two treatment durations: 3/6 days	Winter wheat (Yangmai16, Xumai30)	Decrease	[72]
Booting period	Four temperature levels: day/night, 12/12 h, 2/5 °C, 0/5 °C, −2/5 °C, without Low temperature treatment (CK)	Winter wheat (Wanmai 52), semi-winter (Yannong 19)	Decrease	[81]
Filling period	18/8 °C (day/night), 3 d	Winter wheat (Ningmai 13, Zhenmai 12)	Decrease	[37]
**Starch**—B-type starch granules (%)				
Booting period	Four temperature levels: day/night, 12/12 h, 2/5 °C, 0/5 °C, −2/5 °C, without Low temperature treatment (CK)	Winter wheat (Wanmai 52), semi-winter (Yannong 19)	Decrease	[81]
**Grain traits**—Grain length				
Jointing period, Booting period	Four temperature levels: T_min_/T_avg_/T_max_, 6/11/16 °C (CK), 2/3/8 °C, −4 /1/6 °C, −6/−1/4 °C Three treatment durations: 2/4/6 days	Winter wheat (Yangmai16, Xumai30)	Decrease	[69]
**Grain traits**—Grain width				
Jointing period, Booting period	Four temperature levels: T_min_/T_avg_/T_max_, 6/11/16 °C (CK), 2/3/8 °C, −4 /1/6 °C, −6/−1/4 °C Three treatment durations: 2/4/6 days	Winter wheat (Yangmai16, Xumai30)	Decrease	[69]
Filling period	18/8 °C (day/night), 3 d	Winter wheat (Ningmai 13, Zhenmai 12)	Decrease	[37]
**Grain traits**—Length–width ratio				
Jointing period, Booting period	Four temperature levels: T_min_/T_avg_/T_max_, 6/11/16 °C (CK), 2/3/8 °C, −4 /1/6 °C, −6/−1/4 °C Three treatment durations: 2/4/6 days	Winter wheat (Yangmai16, Xumai30)	Increase	[69]
**Grain traits**—1000 grain weight				
Booting period	Four temperature levels: day/night, 12/12 h, 2/5 °C, 0/5 °C, −2/5 °C, without Low temperature treatment (CK)	Winter wheat (Wanmai 52), semi-winter (Yannong 19)	Decrease	[81]
**Yield**				
Booting period	Four temperature levels: day/night, 12/12 h, 2/5 °C, 0/5 °C, −2/5 °C, without Low temperature treatment (CK)	Winter wheat (Wanmai 52), semi-winter (Yannong 19)	Decrease	[81]
**Protein**—Total protein content (%)				
Jointing period, Booting period	Four temperature levels: T_min_/T_avg_/T_max_, 6/11/16 °C (CK), 2/3/8 °C, −4 /1/6 °C, −6/−1/4 °C Three treatment durations: 2/4/6 days	Winter wheat (Yangmai16, Xumai30)	Increase	[69]
Jointing period, Booting period	Three temperature levels: day/night, 6/16 °C (CK), 0/10 °C, −6/4 °C Two treatment durations: 3/6 days	Winter wheat (Yangmai16, Xumai30)	Increase	[72]
Filling period	18/8 °C (day/night), 3 d	Winter wheat (Ningmai 13, Zhenmai 12)	Increase	[37]
Filling period	−5.5 °C, 3 h	Spring bread wheat (Kariega, SST86), Durum wheat (Oranje), spring soft biscuit wheat (Snack)	Increase	[21]
Filling period	Three temperature levels: day/night, 13/10 °C, 18/15 °C, 23/20 °C	Spring wheat (Avle, Berserk, Bjarne, Zebra)	Decrease	[77]
Filling period	Three temperature levels: day/night, 13/10 °C, 18/15 °C, 23/20 °C	Spring wheat (Bjarne, Cadenza)	Decrease	[76]
**Protein**—Glu/Gli				
Jointing period, Booting period	Four temperature levels: T_min_/T_avg_/T_max_, 6/11/16 °C (CK), 2/3/8 °C, −4 /1/6 °C, −6/−1/4 °C Three treatment durations: 2/4/6 days	Winter wheat (Yangmai16, Xumai30)	Decrease	[69]
**Protein**—UPP (%)				
Filling period	Three temperature levels: day/night, 13/10 °C, 18/15 °C, 23/20 °C	Spring wheat (Cadenza)	Decrease	[76]
Filling period	Three temperature levels: day/night, 13/10 °C, 18/15 °C, 23/20 °C	Spring wheat (Bjarne)	Increase	[76]
**Protein**—Gliadin (%)				
Jointing period, Booting period	Three temperature levels: day/night, 6/16 °C (CK), 0/10 °C, −6/4 °C Two treatment durations: 3/6 days	Winter wheat (Yangmai16, Xumai30)	Increase	[72]
Jointing period, Booting period	Four temperature levels: T_min_/T_avg_/T_max_, 6/11/16 °C (CK), 2/3/8 °C, −4 /1/6 °C, −6/−1/4 °C Three treatment durations: 2/4/6 days	Winter wheat (Yangmai16, Xumai30)	Increase	[69]
**Protein**—ω-gliadin (%)				
Filling period	18/8 °C (day/night), 3d	Winter wheat (Ningmai 13, Zhenmai 12)	Increase	[37]
Filling period	Three temperature levels: day/night, 13/10 °C, 18/15 °C, 23/20 °C	Spring wheat (Bjarne, Cadenza)	Decrease	[76]
Filling period	Three temperature levels: day/night, 13/10 °C, 18/15 °C, 23/20 °C	Spring wheat (Avle, Berserk, Bjarne, Zebra)	Decrease	[77]
**Protein**—α-gliadin (%)				
Filling period	Three temperature levels: day/night, 13/10 °C, 18/15 °C, 23/20 °C	Spring wheat (Avle, Berserk, Bjarne, Zebra)	Increase	[77]
Filling period	Three temperature levels: day/night, 13/10 °C, 18/15 °C, 23/20 °C	Spring wheat (Bjarne, Cadenza)	Increase	[76]
**Protein**—γ-gliadin (%)				
Filling period	Three temperature levels: day/night, 13/10 °C, 18/15 °C, 23/20 °C	Spring wheat (Avle, Berserk, Bjarne, Zebra)	Increase	[77]
Filling period	Three temperature levels: day/night, 13/10 °C, 18/15 °C, 23/20 °C	Spring wheat (Bjarne, Cadenza)	Increase	[76]
**Protein**—Glutenin (%)				
Jointing period, Booting period	Three temperature levels: day/night, 6/16 °C (CK), 0/10 °C, −6/4 °C Two treatment durations: 3/6 days	Winter wheat (Yangmai16, Xumai30)	Increase	[72]
Jointing period	Four temperature levels: T_min_/T_avg_/T_max_, 6/11/16 °C (CK), 2/3/8 °C, −4 /1/6 °C, −6/−1/4 °C Three treatment durations: 2/4/6 days	Winter wheat (Yangmai16, Xumai30)	Increase	[69]
Booting period	Four temperature levels: T_min_/T_avg_/T_max_, 6/11/16 °C (CK), 2/3/8 °C, −4 /1/6 °C, −6/−1/4 °C Three treatment durations: 2/4/6 days	Winter wheat (Yangmai16, Xumai30)	Decrease	[69]
**Protein**—HMW-GS (%)				
Filling period	Three temperature levels: day/night, 13/10 °C, 18/15 °C, 23/20 °C	Spring wheat (Avle, Berserk, Bjarne, Zebra)	unchanged	[77]
Filling period	18/8 °C (day/night), 3 d	Winter wheat (Ningmai 13, Zhenmai 12)	Increase	[37]
**Protein**—D-type LMW-GS (%)				
Filling period	Three temperature levels: day/night, 13/10 °C, 18/15 °C, 23/20 °C	Spring wheat (Avle, Berserk, Bjarne, Zebra)	Decrease	[77]
Filling period	Three temperature levels: day/night, 13/10 °C, 18/15 °C, 23/20 °C	Spring wheat (Bjarne, Cadenza)	Decrease	[76]
**Protein**—B-type LMW-GS (%)				
Filling period	Three temperature levels: day/night, 13/10 °C, 18/15 °C, 23/20 °C	Spring wheat (Avle, Berserk, Bjarne, Zebra)	Increase	[77]
Filling period	Three temperature levels: day/night, 13/10 °C, 18/15 °C, 23/20 °C	Spring wheat (Bjarne, Cadenza)	Increase	[76]
**Protein**—Albumin (%)				
Jointing period, Booting period	Three temperature levels: day/night, 6/16 °C (CK), 0/10 °C, −6/4 °C Two treatment durations: 3/6 days	Winter wheat (Yangmai16, Xumai30)	Increase	[72]
Jointing period, Booting period	Four temperature levels: T_min_/T_avg_/T_max_, 6/11/16 °C (CK), 2/3/8 °C, −4 /1/6 °C, −6/−1/4 °C Three treatment durations: 2/4/6 days	Winter wheat (Yangmai16, Xumai30)	Increase	[69]
**Protein**—Globulin (%)				
Jointing period, Booting period	Three temperature levels: day/night, 6/16 °C (CK), 0/10 °C, −6/4 °C Two treatment durations: 3/6 days	Winter wheat (Yangmai16, Xumai30)	Increase	[72]
Jointing period, Booting period	Four temperature levels: T_min_/T_avg_/T_max_, 6/11/16 °C (CK), 2/3/8 °C, −4 /1/6 °C, −6/−1/4 °C Three treatment durations: 2/4/6 days	Winter wheat (Yangmai16, Xumai30)	Increase	[69]
**Quality traits**—Maximum resistance to extension (Rmax)				
Filling period	Three temperature levels: day/night, 13/10 °C, 18/15 °C, 23/20 °C	Spring wheat (Avle, Berserk, Bjarne, Zebra)	Increase	[77]
**Quality traits**—SDS sedimentation value				
Jointing period, Booting period	Four temperature levels: T_min_/T_avg_/T_max_, 6/11/16 °C (CK), 2/3/8 °C, −4 /1/6 °C, −6/−1/4 °C Three treatment durations: 2/4/6 days	Winter wheat (Yangmai16, Xumai30)	Increase	[69]
Filling period	−5.5 °C, 3 h	Spring bread wheat (Kariega, SST86), Durum wheat (Oranje), spring Soft biscuit wheat (Snack)	Decrease	[21]
**Quality traits**—Wet gluten, Dry gluten				
Jointing period, Booting period	Four temperature levels: T_min_/T_avg_/T_max_, 6/11/16 °C (CK), 2/3/8 °C, −4 /1/6 °C, −6/−1/4 °C Three treatment durations: 2/4/6 days	Winter wheat (Yangmai16, Xumai30)	Increase	[69]
**Quality traits**—Gluten strength				
Filling period	17 to 18 °C	Spring wheat (Avle, Bastian, Bjarne, Zebra)	Decrease	[78]

^1^ The change occurs with a decrease in temperature.

## Data Availability

No new data were created or analyzed in this study. Data sharing is not applicable to this article.
